# Successful Total Knee Arthroplasty in a Patient With Contralateral Ankylosis Due to Severe Heterotopic Ossification

**DOI:** 10.7759/cureus.24941

**Published:** 2022-05-12

**Authors:** Jong Hyun Choi, Benjamin Levens, Jana Fox, Eli Kamara

**Affiliations:** 1 Orthopaedic Surgery, Montefiore Medical Center/Albert Einstein College of Medicine, Bronx, USA; 2 Radiation Oncology, Montefiore Medical Center/Albert Einstein College of Medicine, Bronx, USA

**Keywords:** revision knee arthroplasty, knee replacement, tka, total knee arthroplasty, heterotopic ossification

## Abstract

A 63-year-old woman with ankylosis of the left knee due to severe heterotopic ossification (HO) following total knee arthroplasty (TKA) underwent right TKA with preoperative radiation and postoperative chemical prophylaxis for HO. At the one-year follow-up, the patient had no evidence of HO in the right knee. To our knowledge, there are no reports of successful arthroplasty in patients with a history of ankylosis due to severe HO. We present the first case of successful TKA in a patient with ankylosis of the contralateral knee. TKA can be safely performed in patients at high risk for developing ankylosis.

## Introduction

Heterotopic ossification (HO), although rare, can be a devastating postoperative outcome. HO is the formation of lamellar bone in soft tissue due to the conversion of progenitor cells to osteoprogenitor cells [1]. The exact etiology of HO is unknown, but it is a well-described complication after total knee arthroplasty (TKA) associated with its surgical techniques [2,3]. The overall incidence of HO following TKA has been reported to be up to 42%, and HO after TKA in patients with pre-existing HO has been reported to be as high as 73% [4,5]. Clinical symptoms of HO such as pain, snapping, and stiffness are seen in less than 1% of patients, with incidences of ankylosis limited to case reports [3,6-11]. We present a case of successful TKA in a patient who developed ankylosis in the contralateral knee following surgery. The patient consented to the publication of this case report.

## Case presentation

A 63-year-old woman with a medical history of obesity and osteoarthritis presented to our institution with bilateral knee pain and severe left knee stiffness. Four years prior, she underwent a left TKA at an outside institution for osteoarthritis; office notes, operative reports, and imaging reports were reviewed. A standard anterior approach with medial parapatellar arthrotomy was performed. One year postoperatively, the patient developed worsening pain and dysfunction in the left knee. On examination, her left knee had flexion instability and significant stiffness with a range of motion (ROM) of only 30 degrees. Her X-rays demonstrated severe HO formation medially and posteriorly. The patient was indicated for a left TKA revision. During the revision operation, her left knee was noted to have severe HO involving the medial collateral ligament, which was excised and replaced with a rotating hinge prosthesis to aid in stability. Excess HO and scar tissues were excised in a systemic fashion. No HO prophylaxis was given perioperatively.

Despite her left knee HO excision and revision, the patient continued to complain of pain and stiffness postoperatively. Her ROM was limited to 10-30 degrees. She underwent manipulation under anesthesia (MUA), which improved her ROM intraoperatively to 0-115 degrees. One year after MUA, her ROM again diminished to 30 degrees of flexion. X-rays demonstrated recurrence of extensive HO anteriorly, posteriorly, and medially. The patient underwent another left TKA revision with HO and scar excision. An incision was made through the previous midline approach, and a medial parapatellar arthrotomy was performed. However, due to the excessive scar tissue and HO, the left knee was unable to be flexed to disengage the locking mechanism, and the revision was aborted to prevent further issues.

**Figure 1:**
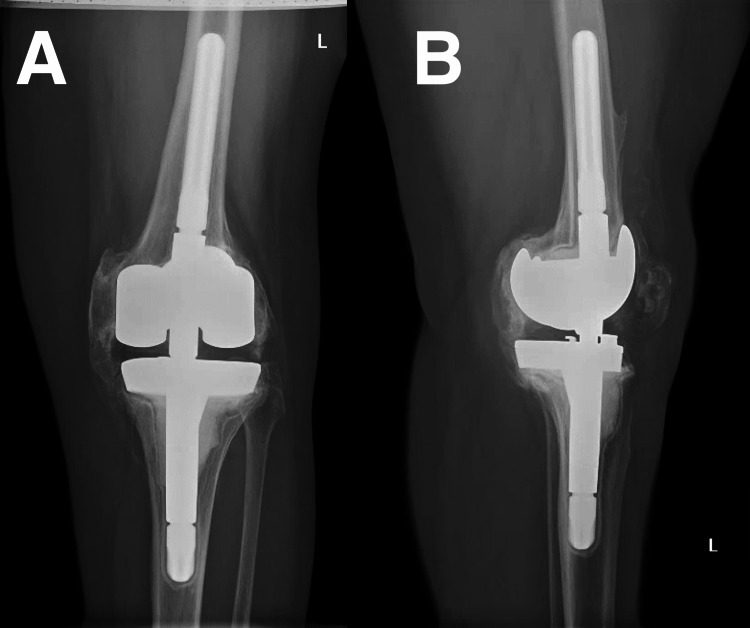
Anteroposterior (A) and lateral (B) radiographs of the left knee demonstrating heterotopic ossification medially, laterally, and posteriorly.

On examination at our institution, the patient’s left knee was fully extended with 0 degrees of motion, and her right knee permitted 0-110 degrees of motion. X-rays demonstrated prior left TKA with ankylosis: medial and posterior bone bridging between the tibia and femur (Figure 1).

**Figure 2:**
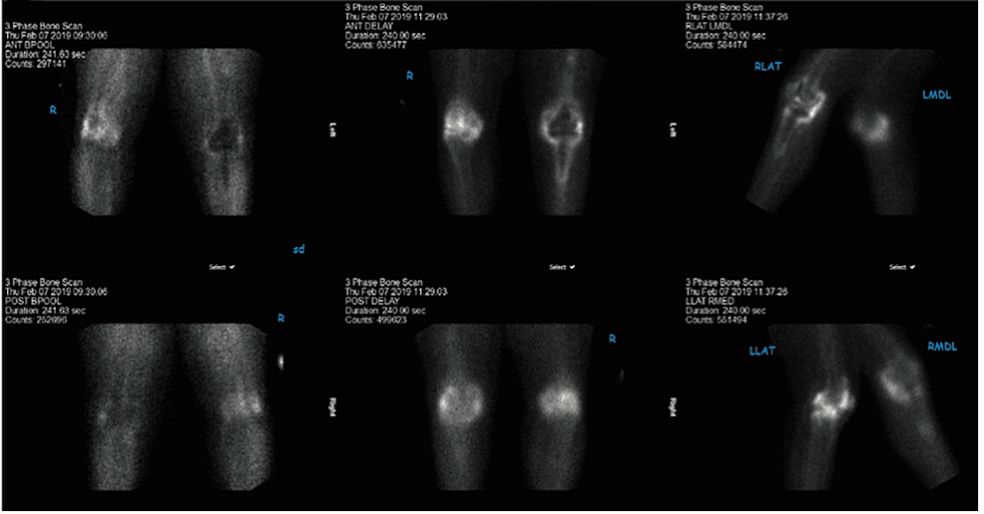
Three-phase bone scan of the bilateral knees demonstrating active uptake in the heterotopic ossification surrounding the left knee prosthesis, medial aspect greater than lateral aspect.

Given her severe pain and stiffness in the left knee, she was indicated for a left knee revision pending further HO workup. Nuclear medicine bone scan was obtained and demonstrated active HO in the left knee (Figure 2).

This was two years following the most recent revision and five years since the original procedure. Given the active HO, it was too early to proceed with the left knee revision and a decision was made to postpone.

**Figure 3:**
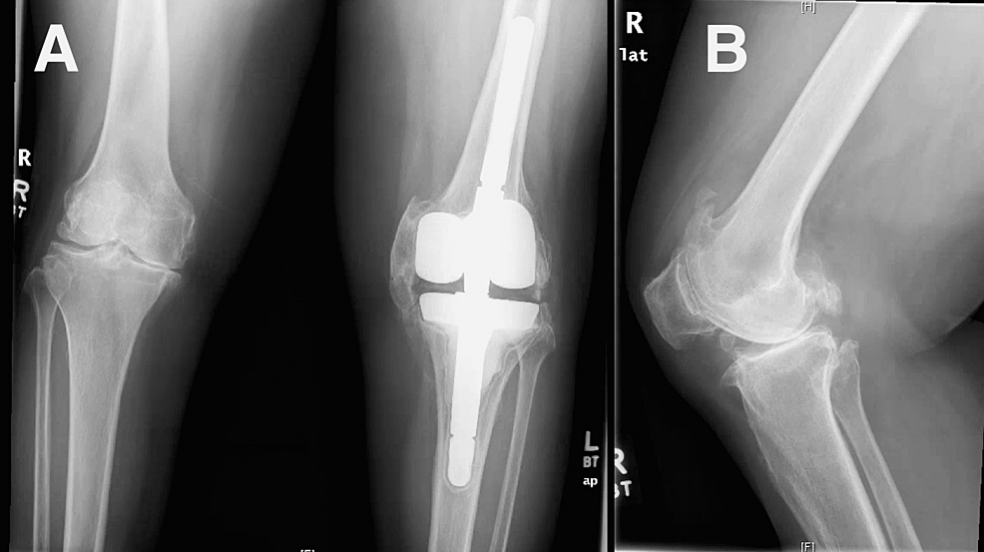
Preoperative bilateral anteroposterior (A) and right lateral (B) radiographs demonstrating osteoarthritis.

The patient complained of worsening right knee pain and expressed her interest in undergoing right TKA, having failed conservative management. Her right knee radiographs demonstrated severe osteoarthritis (Figure 3).

A thorough discussion was had with the patient about the risk of ankylosis postoperatively in the right knee given her history. The patient understood the risks, benefits, and alternatives to surgery; given her failure to respond to conservative therapy, she consented to proceed with the right TKA.

**Figure 4:**
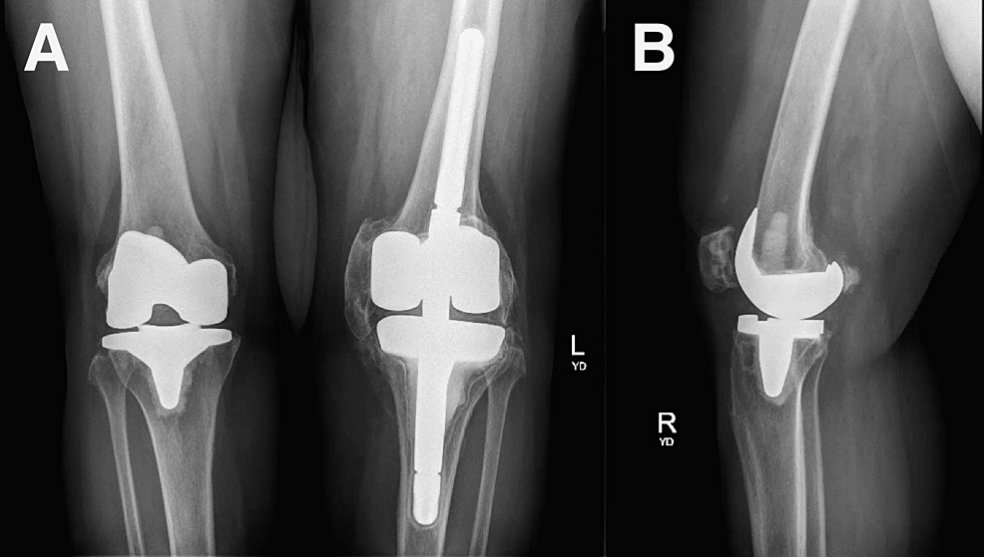
One-year postoperative bilateral anteroposterior (A) and right lateral (B) knee radiographs demonstrating no evidence of heterotopic ossification in the right knee.

To minimize the risk of postoperative HO in her right knee, a regimen of preoperative radiation and postoperative indomethacin and etidronate was planned. One day prior to the surgery, the patient received a single dose of 7 Gray (Gy) to the periarticular region, delivered via anterior and posterior fields. Intraoperatively, a standard anterior approach to the right knee was performed with medial parapatellar arthrotomy. The surgeon’s standard TKA procedure was performed uneventfully. Postoperatively, she started on indomethacin 25 mg three times a day (TID) for three months. She progressed well with therapy and was discharged home on postoperative day two. Twelve days postoperatively, the patient was seen in the office with improved postoperative right knee pain. On examination, her ROM of the right knee was 5-115 degrees. One month postoperatively, the patient returned to her normal activities. On examination, her ROM of the right knee was 0-110 degrees. X-rays did not demonstrate any evidence of HO in her right knee. The patient continued to be compliant with indomethacin 25 mg TID, and etidronate 400 mg four times a day (QD) for three months was added to her HO prophylaxis regimen. Two months postoperatively, her ROM of the right knee remained unchanged. Three months postoperatively, indomethacin 25 mg TID was discontinued, and etidronate 400 mg QD was discontinued the month after. Six months postoperatively, the patient’s ROM of the right knee was 0-110 degrees. At the one-year follow-up, X-rays demonstrated well-fixed and aligned right TKA with no evidence of HO in the right knee (Figure 4).

Her ROM of the left knee was 0 degrees with no flexion and the right knee was 0-120 degrees. Nuclear medicine bone scan continued to demonstrate active HO in the left knee without evidence of HO in the right knee.

## Discussion

Incidence of HO after TKA in patients with pre-existing HO remains elusive, with the only published study by Furia and Pellegrini that demonstrated eight of eleven (73%) patients with pre-existing HO at unspecified locations developed HO near the site of TKA [4]. Ankylosis secondary to HO after TKA is rare, and its effects on the arthroplasty of the contralateral knee are not known. Cases of ankylosis due to HO after TKA are limited, and reports of arthroplasty of the contralateral knee are not available in the literature [7-11].

Predictors of HO following TKA include pre-existing HO, hypertrophic arthrosis, and increased lumbar bone mineral density [4,12]. Surgical techniques of TKA such as femoral notching, postoperative hematoma/effusion, trauma to the quadriceps tendon, and excessive soft-tissue retraction may predispose patients to HO [2,3]. HO following TKA commonly presents in the anterior aspect of the distal femur but can also present laterally and posteriorly [2-4,6,12-14]. HO can be assessed clinically with postoperative ROM and quantitatively with anteroposterior and lateral radiological views within three to four weeks after surgery [1]. Nuclear bone scans may aid in evaluating the maturation process. There is no standardized or validated classification system for HO after TKA that is similar to the Brooker classification, which is widely adopted to classify HO after THA. Several classifications based on anteroposterior and lateral radiological views have been proposed; however, most fail to consider clinical function or indications for surgery [1,2,4,5,12]. Given the low likelihood of clinical symptoms of HO after TKA, ankylosis of the knee is rare and limited to case reports [3,7-11]. The rarity of severe cases may be attributed to the spontaneous resolution of clinical symptoms such as restricted ROM and pain [2,15].

Prophylaxis with preoperative radiation, postoperative indomethacin, or combination radiation and indomethacin therapy are effective against HO formation after THA. However, there is a paucity of literature on HO prophylaxis after TKA compared to studies on HO prophylaxis after THA. Recommended prophylaxis of HO after TKA is based on THA studies. One of the first published cases on HO prophylaxis of the knee is by Lo et al., who reported successful radiation prophylaxis with 7 Gy after revision TKA in a patient who developed symptomatic HO following initial TKA [16]. Subsequent studies reported no recurrence of HO with 7 Gy of postoperative radiation in high-risk patients and those who underwent HO excision [6,17]. Some studies have proposed non-steroidal anti-inflammatory drug use as attributing factors to the low incidence of HO after TKA [12,15]. Although not specific to arthroplasty, bisphosphonates have been shown to halt the progression of HO after early diagnosis in spinal cord injury and hasten the maturity of HO in burn patients with minimal adverse effects [18,19]. Clinical studies on HO and its prophylaxis following TKA in patients with pre-existing HO of the contralateral knee are limited and remain to be elucidated. HO prophylaxis is recommended for high-risk patients undergoing TKA.

## Conclusions

To our knowledge, this is the first case report of successful TKA in a patient with ankylosis due to severe HO in the contralateral knee. Given the high risk of developing HO after TKA, the patient received 7 Gy of radiation one day preoperatively, indomethacin 25 mg, and etidronate 400 mg for three months postoperatively to prevent HO formation. The combination of prophylactic therapy resulted in a successful TKA outcome without any clinical or radiographic signs of HO at one-year postoperatively.
